# Supplementary material for: Complexome profiling of the Chlamydomonas psb28 mutant reveals TEF5 as an early PSII assembly factor
Source: Plant Cell. 2025 Mar 17;37(6):koaf055. doi: 10.1093/plcell/koaf055 (PMC12164590; doi:10.1093/plcell/koaf055)
Supplement: koaf055_Supplementary_Data [file koaf055_supplementary_data.zip › psb28_interactive.zip!/plots/intensity/qualityPlots/peptides/_Cre06.g278222.t1.1.html]

### Cre06.g278222.t1.1

Trivial name:   
SeqCoverage [median]: 15.40  
Mass: 35.14 kDa  
  
 

| Sequence | MissedCleavage | Mass [Da] | wtIntensity | muIntensity | Score |
| --- | --- | --- | --- | --- | --- |
| AETLTLR | 0 | 802.45 | 565.03 | 0.00 | 91.91 |
| SESNYGYAR | 0 | 1045.45 | 775.97 | 0.00 | 74.13 |
| SIVDDLRPEFNITSK | 1 | 1732.90 | 1071.00 | 0.00 | 28.30 |
|  |  |  | 2412.0 | 0.0 |  |
